# Cause rare d'une toux sèche à l'effort: agénésie de l'artère pulmonaire gauche avec hypoplasie pulmonaire

**DOI:** 10.11604/pamj.2017.27.146.11399

**Published:** 2017-06-29

**Authors:** Azzeddine Laaraje, Naima El Hafidi, Chafik Mahraoui

**Affiliations:** 1 Service de Pneumo-Allergologie, Hôpital d'Enfants, CHU Avicenne, Rabat, Maroc

**Keywords:** Agénésie de l´artère pulmonaire, hypoplasie pulmonaire, toux d´effort, Agenesis of the pulmonary artery, pulmonary hypoplasia, exertional cough

## Abstract

L'agénésie de l'artère pulmonaire gauche associée à l'hypoplasie du poumon homolatéral, est une malformation congénitale rare qui peut être découverte chez l'enfant de façon fortuite ou par des infections respiratoires récidivantes. Son diagnostic est établi par l'angioscanner thoracique. Le traitement est essentiellement conservateur. Nous rapportons un cas survenant chez un enfant de 6 ans et révélé par une toux d'effort.

## Abstract (en)

Agenesis of the left pulmonary artery associated with hypoplasia of the ipsilateral lung is a rare congenital malformation in children; it can be discovered fortuitously or because of the presence of recurrent respiratory infections. Diagnosis is based on thoracic angioscanner. Treatment is essentially conservative. We report the case of a 6-year old child with agenesis of the left pulmonary artery associated with hypoplasia of the ipsilateral lung detected because of exertional dry cough.

## Introduction

L'agénésie unilatérale de l'artère pulmonaire est une malformation congénitale rare, pouvant être la source de complications graves. Son diagnostic doit être évoqué devant des infections respiratoires récidivantes. L'angioscanner thoracique permet de visualiser l'agénésie de l'artère pulmonaire et les anomalies parenchymateuses associées. La prise en charge de ces patients n'est pas consensuelle à ce jour et dépend de l'évolution clinique.

## Patient et observation

**Figure 1:**
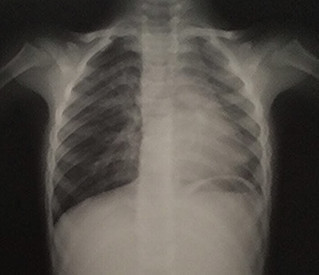
Radiographie thoracique montrant une asymétrie de taille des deux champs pulmonaire avec un foyer basal

**Figure 2:**
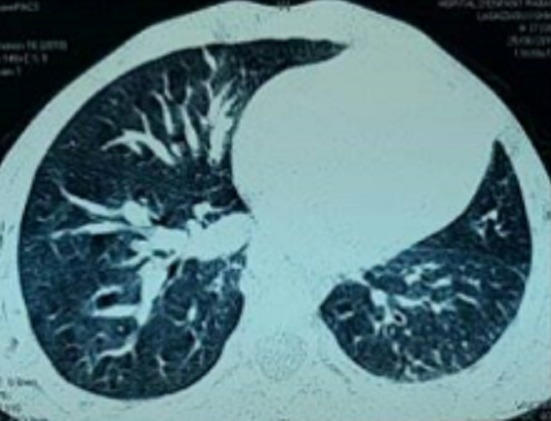
Hypoplasie pulmonaire gauche visualisée sur le scanner thoracique

**Figure 3:**
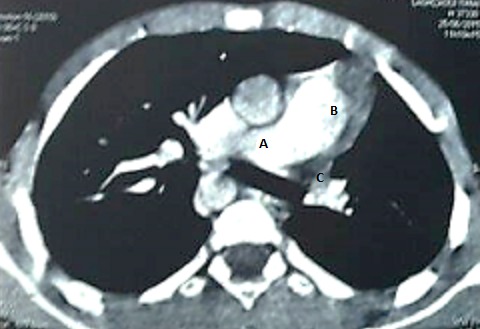
Angioscanner thoracique objectivant l'agénésie de l'artère pulmonaire gauche avec hypoplasie du poumon gauche: (A) origine de l'artère pulmonaire droit; (B) tronc de l'artère pulmonaire: (C) absence d'individualisation de structure vasculaire sur le trajet de l'artère pulmonaire gauche: agénésie

**Figure 4:**
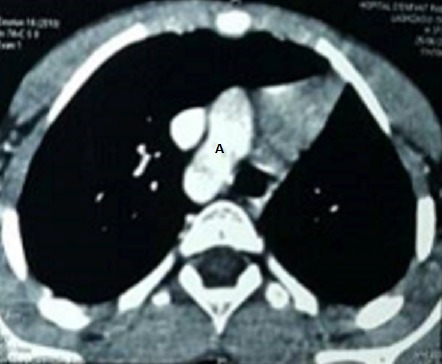
Arc aortique droit sur l'angioscanner thoracique

Il s'agit d'un enfant de 6 ans, de sexe masculin, ayant comme antécédents des infections respiratoires à répétition et une consanguinité de premier degré, il n'y a pas de notion de contage tuberculeux. L'histoire de sa maladie remonte à l'âge de 3 mois par une toux sèche, diurne et nocturne, sans cyanose ni hémoptysie, évoluant dans un contexte de fièvre intermittente et de conservation de l'état général. L'enfant a été traité en ambulatoire à plusieurs reprises. Devant la persistance de cette toux, survenant essentiellement lors de l'effort, l'enfant a été adressé au service de pneumo-allergologie de l'hôpital d'enfant de Rabat pour exploration. A l'examen clinique, l'enfant était eupnéique, présentant un retard pondéral (-2 déviation standard) et un hippocratisme digital. À l'auscultation, on a noté un souffle systolique et une diminution du murmure vésiculaire au niveau de l'hémi-champ pulmonaire gauche. Les pouls fémoraux étaient présents et symétriques. Par ailleurs, on n'a pas décelé d'hépatosplénomégalie. La radiographie thoracique a objectivé une asymétrie de taille des deux champs pulmonaire, une distension du poumon droit avec un foyer basal et un syndrome bronchique (Figure 1). Les lésions étaient non progressives sur les clichés thoraciques. L'échocardiographie n'a pas montré d'anomalie cardiaque. Le scanner thoracique a objectivé une hypoplasie du poumon gauche (Figure 2). On a complété le bilan par une angioscanner thoracique qui a révélé l'agénésie de l'artère pulmonaire gauche avec hypoplasie du poumon gauche alimenté par des branches collatérales fines (Figure 3) associé à un arc aortique droit (Figure 4). Un bilan phtysiologique (intradermo réaction à la tuberculine, recherche de bacille de Koch dans les crachats) était négatif. La pH-métrie est revenue normale. Le diagnostic d'une toux d'effort sur agénésie de l'artère pulmonaire gauche avec hypoplasie du poumon gauche a été retenu. L'enfant a été mis sous traitement conservateur: antibiothérapie et kinésithérapie respiratoire avec surveillance clinique, radiologique et échocardiographique rigoureuse.

## Discussion

L'agénésie unilatérale primitive d'une artère pulmonaire (AUAP) est une malformation congénitale rare. Décrite initialement par Fraentzel en 1868, c'est seulement en 1952 qu'elle est visualisée sur une angiographie par Madoff et al [1]. Son incidence est d'environ 1 sur 200,000 et ne représente que 1% des cardiopathies congénitales [2, 3]. Pour Sherrick et al, l'agénésie est plus fréquente du côté droit que gauche. De plus, 58% des agénésies de l'artère pulmonaire droite sont isolées contre seulement 19% des agénésies de l'artère pulmonaire gauche [4]. Sur le plan embryologique, les branches de l'artère pulmonaire sont dérivées de la portion proximale des sixièmes arcs aortiques primitifs. La disparition de la portion proximale de l'arc droit ou gauche entraîne l'absence de formation d'un artère pulmonaire [5]. L'hypoplasie pulmonaire homolatérale s'explique par le parallélisme entre le développement vasculaire et la croissance alvéolaire: l'arrêt de croissance d'une artère pulmonaire entraîne un défaut de croissance alvéolaire périphérique à l'origine d'une hypoplasie pulmonaire harmonieuse diffuse [6]. Elle est rarement symptomatique, découverte la plupart du temps dans l'enfance suite à des complications ou dans le cadre d'un syndrome malformatif cardio-vasculaire. La tétralogie de Fallot, la communication interventriculaire, l'arc aortique droit, la transposition des gros vaisseaux, un retour veineux anormal ou la persistance d'un canal artériel sont les principales malformations cardiovasculaires associées [7]. Les complications possibles sont les infections respiratoires récidivantes (37%), la dyspnée ou une gêne à l'effort (40%), l'hémoptysie (20%), l'hypertension artérielle pulmonaire (25%) et l'œdème aigu cardiogénique du poumon [8]. Dans notre cas, l'enfant présentait des infections respiratoires récidivantes, une toux chronique s'aggravant à l'effort. On a également découvert un arc aortique droit associé. Boudard et al rapportent dans leur étude la grande fréquence de patients présentant une symptomatologie pouvant évoquer un asthme(75%) comme une toux sèche à l'effort, parfois associée à des sibilants.

La radiographie thoracique peut retrouver différents signes qui permettront d'évoquer le diagnostic: une asymétrie de vascularisation, une absence d'ombre hilaire, un petit poumon hyperclair avec attraction du médiastin, parfois un emphysème compensateur du poumon controlatéral. Le diagnostic était classiquement réalisé par angiographie, mais l'angioscanner reste l'examen de référence, il montre l'absence de structure vasculaire sur le trajet de l'artère pulmonaire avec une paroi lisse et régulière entre l'artère pulmonaire primitive et l'artère pulmonaire droite ou gauche restante et permet également d'objectiver une éventuelle hypoplasie associée [8], retrouvée chez notre enfant. L'analyse des veines pulmonaires par l'angioscanner élimine d'emblée une embolie pulmonaire ou un syndrome du cimeterre, des pathologies qui peuvent être associée à une séquestration pulmonaire [8]. L'angiographie à visée thérapeutique reste indiquer en cas d'hémoptysie. L'échographie cardiaque est utile dans le dépistage d'autres malformations cardiaques associées. Elle est indispensable au suivi et permet de vérifier l'absence de survenue d'une hypertension artérielle pulmonaire. L'intérêt de la scintigraphie pulmonaire de ventilation-perfusion réside dans l'évaluation de la fonctionnalité du poumon et permet d'orienter une éventuelle thérapeutique. La prise en charge se limite souvent à la surveillance: clinique, fonction respiratoire avec épreuve d'effort et échographie cardiaque. Elle doit être adaptée au cas par cas en fonction des complications présumées: traitement des infections respiratoires récidivantes, vaccination. La pneumonectomie et l'embolisation restent des alternatives thérapeutiques devant des manifestations importantes à l'effort ou encore des hémoptysies et relèvent d'une décision multidisciplinaire.

## Conclusion

L'agénésie de l'artère pulmonaire gauche avec hypoplasie pulmonaire est une malformation rare dont le pronostic peut être fatal. Souvent asymptomatique, elle peut être révélée par des complications: infection respiratoire à répétition, toux, dyspnée d'effort et hémoptysie. L'angioscanner permet de poser le diagnostic. Ces patients nécessitent un suivi attentif (clinique et échocardiographique) compte tenu des risques d'hypertension artérielle pulmonaire et d'hémoptysie.

## Conflits d'intérêts

Les auteurs ne déclarent aucun conflit d'intérêt.
